# Development and verification of an agent-based model of opinion leadership

**DOI:** 10.1186/s13012-014-0136-6

**Published:** 2014-09-27

**Authors:** Christine A Anderson, Marita G Titler

**Affiliations:** School of Nursing, University of Michigan, Ann Arbor, MI USA

## Abstract

**Background:**

The use of opinion leaders is a strategy used to speed the process of translating research into practice. Much is still unknown about opinion leader attributes and activities and the context in which they are most effective. Agent-based modeling is a methodological tool that enables demonstration of the interactive and dynamic effects of individuals and their behaviors on other individuals in the environment. The purpose of this study was to develop and test an agent-based model of opinion leadership. The details of the design and verification of the model are presented.

**Methods:**

The agent-based model was developed by using a software development platform to translate an underlying conceptual model of opinion leadership into a computer model. Individual agent attributes (for example, motives and credibility) and behaviors (seeking or providing an opinion) were specified as variables in the model in the context of a fictitious patient care unit. The verification process was designed to test whether or not the agent-based model was capable of reproducing the conditions of the preliminary conceptual model. The verification methods included iterative programmatic testing (‘debugging’) and exploratory analysis of simulated data obtained from execution of the model. The simulation tests included a parameter sweep, in which the model input variables were adjusted systematically followed by an individual time series experiment.

**Results:**

Statistical analysis of model output for the 288 possible simulation scenarios in the parameter sweep revealed that the agent-based model was performing, consistent with the posited relationships in the underlying model. Nurse opinion leaders act on the strength of their beliefs and as a result, become an opinion resource for their uncertain colleagues, depending on their perceived credibility. Over time, some nurses consistently act as this type of resource and have the potential to emerge as opinion leaders in a context where uncertainty exists.

**Conclusions:**

The development and testing of agent-based models is an iterative process. The opinion leader model presented here provides a basic structure for continued model development, ongoing verification, and the establishment of validation procedures, including empirical data collection.

## Background

To improve patient outcomes and the provision of care based on research evidence, it is critical that we speed up and optimize the process of translating evidence from research into practice. Use of opinion leaders (OLs) is one implementation strategy suggested to decrease the research to practice gap. Opinion leaders are from the local peer group, viewed as a respected source of influence, considered by associates as technically competent, and trusted to judge the fit between the evidence base of the practice and the local situation [1-3]. Opinion leadership is multifaceted and complex, with role functions varying by the circumstances, but few successful projects to implement innovations in healthcare organizations have managed without opinion leaders [4-6]. Although use of opinion leaders improves practice performance, much is still unknown about the best methods of selecting opinion leaders, specific attributes of opinion leaders, actual activities opinion leaders use to improve practice, and the context/setting (acute versus primary care) in which OLs are most effective [2].

Agent-based modeling is a methodological tool that enables demonstration of the interactive and dynamic effects of heterogeneous individuals and their behaviors on other individuals in their environment. Agent-based models (ABMs) are useful to simulate theorized relationships and thereby contribute to theory development. Analysis of data obtained from simulations may lead to further elaboration or revision of a theory prior to the collection of actual empirical data [7]. Actual data, once obtained, can then be used to further refine and test the model [8]. According to Epstein, ABM facilitates the ability to ‘generate’ a phenomenon of interest, which contributes to explanation in social science [9-11]. The overall purpose of this study was to develop and test an agent-based model of opinion leadership in nursing. The aims of representing both the contextual and dynamic nature of opinion leadership led to the use of this methodological tool [12]. Verification of the ABM, the process of testing correspondence with the underlying conceptual model, is a key step toward using the model to gain new insights and generate new questions about opinion leadership and to guide validation efforts such as empirically testing the model via research.

**Figure 1:**
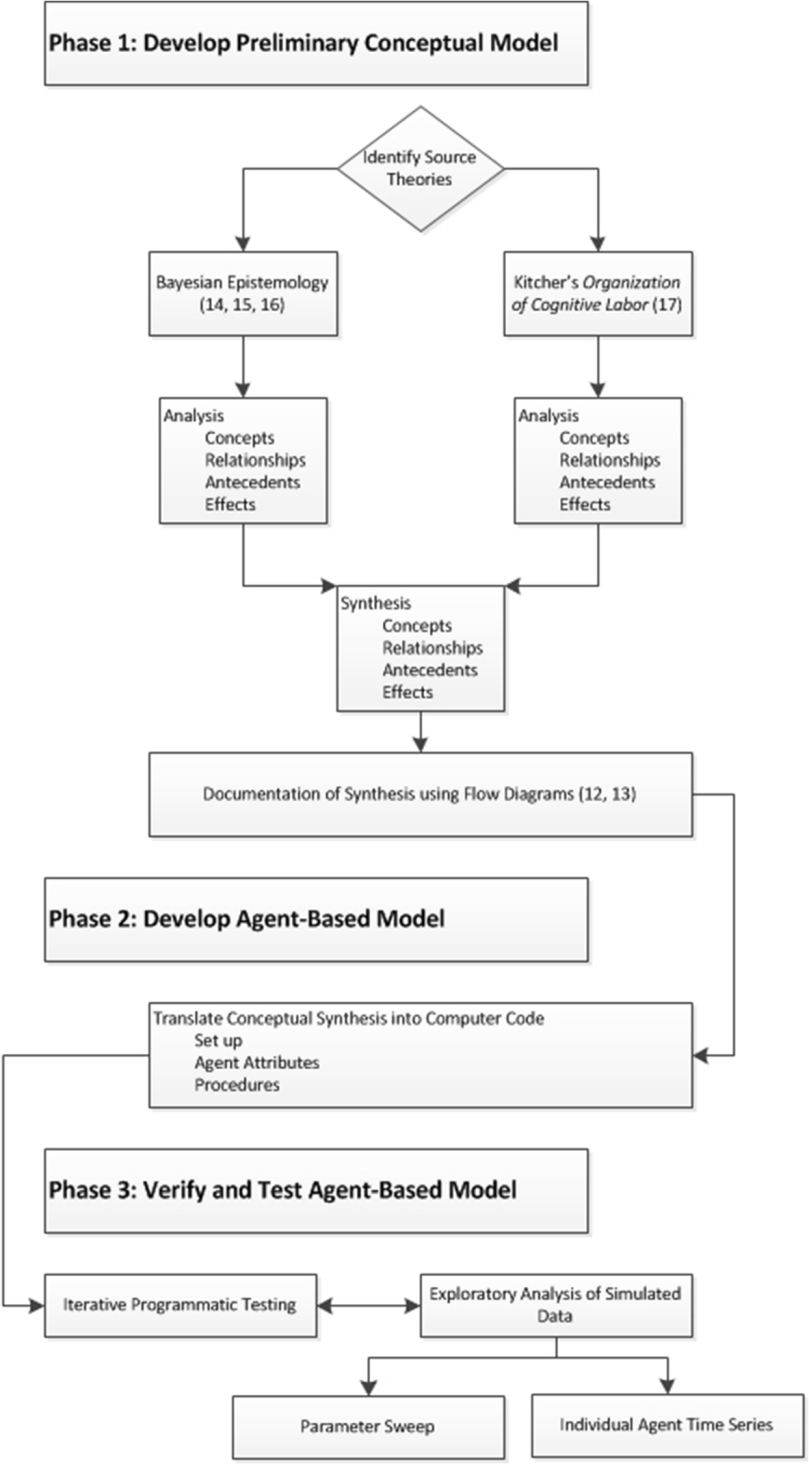
**Flow chart of study methods.** This figure depicts the three phases of the overall modeling study. Phases 2 and 3 are the focus of this report.

The development of the nurse opinion leader agent-based model (NOL-ABM) involved three phases of work: 1) development of the preliminary conceptual model of NOL; 2) designing the NOL-ABM by translating the concepts, specifications, and processes defined in the preliminary NOL model into computer code; and 3) verifying the NOL-ABM though programmatic testing and analysis (Figure 1). Phase 1, the development of the preliminary NOL model, is described in detail elsewhere [12,13] with a brief overview provided herein. The focus of this paper is to describe the details of the design (phase 2) and verification testing (phase 3) of the NOL-ABM model.

## Methods

### Overview of preliminary conceptual NOL model development

The development of the preliminary model is described in detail elsewhere [12,13]; however, a basic overview is provided here for clarity related to the process of ABM development. The preliminary model of NOL is a normative (rather than empirical) model of nursing opinion leadership derived from philosophic theories about belief formation [12,13]. Two source theories, Bayesian epistemology as described by Joyce [14-16] and Kitcher’s *Organization of Cognitive Labor* [17] were selected because they examine the basis for opinion formation in individuals (Joyce) and groups (Kitcher). Using theory derivation and synthesis methods developed by Walker and Avant [18], each of the two theories was analyzed to identify concepts, relational statements, antecedents, and effects. These components were then synthesized, in order to create a representation of opinion leadership in nursing, for use as a guide to computer programming for the ABM (Table 1). The NOL model explains the dynamic and multi-level phenomenon of how the opinions and actions of individual nurses affect the beliefs and practice behaviors of others from the same community (e.g. patient care unit or hospital). The model also addresses contextual factors that contribute to the emergence of nurse opinion leaders within the community over time. These factors include the size of the group, the degree of uncertainty among individual group members regarding evidence, and the availability of motivated and credible individuals who can act as NOL [13]. For example, if a new method for preventing patient falls is introduced on a patient care unit, individual nurses may evaluate the practice and adopt it. Some nurses may be uncertain that the evidence is credible and, rather than spend time investigating on their own, they may ask another nurse, who is believed to be credible, for an opinion. The extent to which such an opinion influences behavior varies depending on the relationship between the co-workers and the strength of belief regarding current practice. The simple request for advice by one nurse to a co-worker does not necessarily indicate the presence of an opinion leader. When multiple individuals seek out the same person for advice, repeatedly and over time, the potential for opinion leadership exists. Next, the methods used to design and test the NOL-ABM, based on the concepts and relationship identified in this phase, are described.

**Table 1:** **NOL-ABM variables**

**Variable** ^**a**^	**Specifications**
*Global variables*	*Values can be accessed by all agents*
*Announced evidence*—new evidence made known to agents, expressed as a probability	Value (1–100) based on a random normal distribution around a mean determined by the model user, visible to the agents
*Credibility of the evidence announcer*—probability that what the announcer says is true	Value (1–100) of the credibility of the random individual agent that announces the evidence, made visible to other agents
*Agent set variables*	*Value determined by membership in a group*
*Unearned authority (UA)—*authority resulting from the agent’s position	Defined by position: UA of staff nurses = 50, UA of educators = 80, UA of nurse managers = 90
*Agent variables*	*Each agent has unique value assigned by the model program based on model user input of the mean*
*Prior-belief*—individual agent’s level of confidence as to the probability of a given proposition	Agent belief at the beginning of process. Initial setting is random normal distribution (1–100) with model user adjusted mean. Sequential values are determined by the belief revision process.
*Earned authority*—authority based on a person’s performance	Random normal distribution (1–100) with model used adjusted mean
*Motives*—probability that an individual takes a course of action based on epistemic (truth) or pragmatic (utility) goals	Random normal distribution (1–100) with model user adjusted mean. <50 = pragmatic, ≥50 = epistemic.
*Procedure-based agent variables*	*Values calculated based on agent procedures*
*Visibility*—agent’s behaviors are made known to others	Prior belief combined with a threshold based on motives. Pragmatic agents have a lower prior-belief threshold for visibility
*Credibility*—evaluation about the probability that what the agent says is true	Weighted combination of earned and unearned authority. Weight based on visibility of agent.
*Assessed evidence—*agent’s evaluation of the truth value of new evidence	Absolute value of the difference between an agent’s prior belief and the announced evidence
*Assessed credibility of announcer*—agent compares his own perceived credibility with that of the announcer	Absolute value of the difference between an agent’s own credibility and credibility of the announcer who shares the new evidence
*Uncertainty*—agent unable to assess the truth value of the evidence	Based on a threshold of evidence and credibility assessments. Determines need for advice
*Availability—*agent meets the threshold requirements to act as an advice giver	Visible agents with a model user adjusted threshold of credibility available for giving opinion to other agents seeking advice
*Get advice—*seek out available agents as a resource to decrease uncertainty about evidence	Agents who need advice create links with available opinion resources (potential OLs). Reassess evidence and announcer credibility based on the beliefs and credibility of the opinion resources
*In-link*—incoming communication from an uncertain agent to an agent available to give advice	Number of links an available agent receives from uncertain agents
*Out-link*—outgoing communication from an uncertain agent	Number of links an uncertain agent sends to available agents
*New belief—*revised probability assessment of the evidence	Agents change their beliefs based on their prior beliefs and a threshold assessment of the evidence. Individual agent’s new belief replaces the prior belief for the next sequence (tick). If the assessment does not meet the threshold for revising belief, the new belief remains the same as the prior belief. Aggregate of individual belief revision changes the overall community context in terms of consensus belief.

^a^Derived from Joyce [14-16] and Kitcher [17] as described in Anderson and Whall [13].

### Overview of the ABM development and verification testing

Following the development of the underlying conceptual NOL model, the steps for developing an ABM begin with the specification and programming of attributes and behaviors of individuals, termed agents, using a software development platform. The developmental process includes verification and testing of the model execution. Once the preliminary verification process is complete, ‘experiments’ are conducted to further verify the model’s performance by statistically analyzing simulated data obtained as output [19]. The following describes the creation of the NOL-ABM using NetLogo [20]. NetLogo, one of several ABM development platforms available, was selected for use in this effort because of its ‘ease of use’ as well as its extensive documentation. We first describe the programming of the basic elements of the ABM, representing nurses (agents) with attributes and behaviors that work on a fictitious nursing unit, followed by the processes used to verify that the computer model represents the concepts and relationships proposed in the preliminary NOL model.

### Agent attributes and behaviors

The individual agent perspective is a central feature of ABM. The development of the NOL-ABM began with specification of individual agent attributes based on the concepts and relationships developed in the preliminary model. Within the Netlogo programming environment, individuals are ‘agents’ and ‘agent sets’ are groups of agents that behave in defined ways. The NOL-ABM contains three agent sets; staff nurses, educators, and nurse managers. Agent-set variables have values determined by membership in the group. For example, Kitcher’s definition of unearned authority, as authority assigned as a result of position (e.g. nurse manager), was used in the preliminary NOL model [13,17]. Therefore, in the ABM, the variable ‘unearned authority’ has a different defined value for each of the three positions that are represented—educators, nurse managers, and staff nurses (See Table 1).

**Figure 2:**
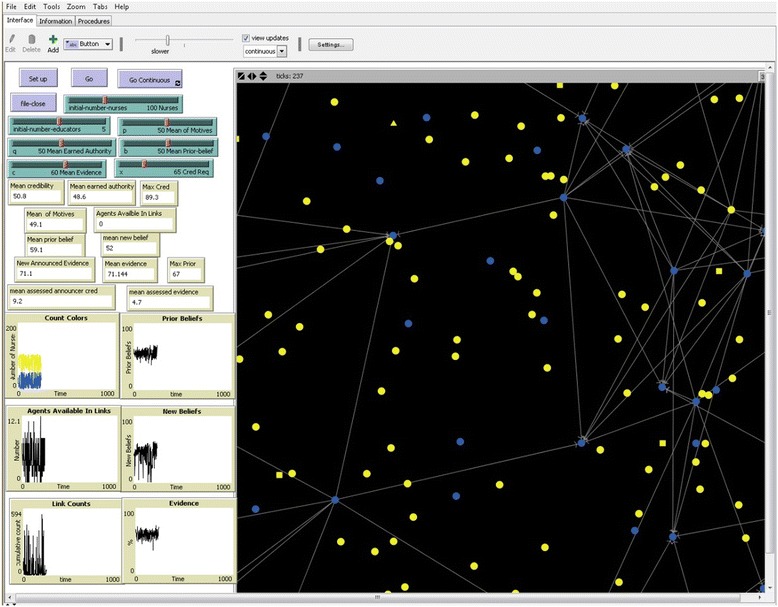
**The NOL-ABM program interface.** This figure is a screenshot of the model interface and shows the various user inputs and graphical displays. The large area on the right of the figure displays the agents and connections among them. The shape and color of the agents represent attributes. The *circles* are staff nurses, *squares* are educators, and *triangle* is the manager. *Blue* represents ‘visible’ agents and *yellow* means the agents are not visible to their colleagues. The *lines* represent links based on requests for opinions based on the visibility and credibility of agents who may become opinion leaders.

By contrast, individual/agent variables, or attributes, are specified so that each agent has his/her own unique value. The value range is variable and randomly assigned, based on the input of the investigator or model user, via adjustments on the model interface, shown in Figure 2. The agents all have their own prior beliefs, earned authority, and motives. The values for these are programmed to be computer generated based on a random normal distribution around an adjustable mean (set by the user on the interface) and a fixed standard deviation. The ‘motive’ variable is determined in this way so that individuals are assigned a random motive value on a scale of 1–100, where motives <50 are considered pragmatic (seeking to maximize best interest) and motives ≥50 are epistemic (seeking to maximize accuracy of beliefs). Adjusting the setting of the mean ‘motives’ allows the user to observe agent behaviors on units that are more or less pragmatic overall. Like the motives variable, the initial prior beliefs and earned authority are randomly set on a scale of 0–100 to reflect probabilities. Using the model interface, the initial mean of all of the agent’s prior beliefs and earned authority are set and then individual agent values are computer generated and randomly assigned to the agents based on the normal distribution. Adjustments to the standard deviation result in more or less variability among the agents.

In addition to the individual agent and agent-set variables, ‘global variables’ are those that have only one value that is accessible by all of the agents. In the NOL-ABM, ‘announced evidence’ is an example of a global variable—all agents can ‘see’ the value when it is announced by a random agent. The credibility of the agent that announces the evidence is also global, that is, it is a value attached to the individual agent and accessible to all of the other agents.

Finally, in addition to possessing attributes, individual agents also perform various behaviors or actions defined in computer code as ‘procedures’. Many of the agent variables in the NOL-ABM are procedure-based, meaning that the values are calculated based on the actions taken by the agents. Table 1 provides the details about the NOL-ABM variables and the specifications for each.

### Programming and execution

The programming of the NOL-ABM was iterative and began with coding the initialization of the ‘setup’ of the model. Initialization includes creating the specified number of agents and assigning values to the attributes (prior belief, earned authority, motives) of each agent. Additionally the setup includes the specification of the visual representation of the group to which each agent belongs—e.g. the staff nurses are ‘circles’ and the educators ‘squares’ (Figure 2).

Following completion of the programming for basic initialization, the next step was to program the execution of the model (i.e. specification of what actually happens when the model runs). When the user clicks on the ‘go’ button on the model interface, behaviors of the agents, such as obtaining new evidence, seeking opinions and updating their beliefs occur. Based on the preliminary NOL, the first step is when the agents obtain new evidence about a given topic. The announcement of new evidence (with a random probability), by a random nurse, on a given unit, to the others on that unit, begins each sequence or ‘tick’. Next, each nurse assesses the evidence and the credibility of its source. Assessment is achieved when the nurses compare their own beliefs and credibility to the new information. For programming purposes, evidence assessment was executed by calculating the difference, in absolute value, of the agent’s prior beliefs and the probability of the new evidence. The credibility assessment of the agents was similarly defined by programmed calculation procedures.

The new evidence is probable (to the nurse) if it is within a specified range of difference from the individual’s own prior belief. The evidence announcer (randomly selected by the program from among the available agents) is also credible, relative to the assessor. In the case of ‘probable evidence,’ the nurses adopt the evidence and revise their beliefs. Programming of the belief revision rule took into account the prior beliefs of the nurse, the evidence, and the credibility of the evidence announcer. The resulting strength of belief, combined with the motives of the individual nurse, determines whether the nurse will act on the belief and therefore become visible to the other nurses. This is important since, in order to be available as an opinion resource, the nurse agent must be willing to act.

After each evidence assessment, if nurses are uncertain about either the evidence, or the credibility of the announcer, they may seek advice from other credible nurses with visible beliefs. If individuals are available (e.g. credible and visible) to act as opinion resources, the uncertain nurses may adjust their assessments based on the beliefs and credibility of the nurse whose advice was sought. Following reassessment, reapplication of the decision rule regarding adoption of new evidence occurs, and beliefs are revised if indicated. See Table 2 for a summary of the procedures performed by the agents in the NOL-ABM.

**Table 2:** **Agent procedures**

**Procedure group**	**Individual procedures**
*Initialization (initial set up of model parameters)*	Create agents (nurses, educators, managers)
	Set unearned authority
	Set prior belief
	Set earned authority
	Set motives
	Set visibility
	Set credibility
*To go (start sequence of events)*	Announce evidence (one of agents is an announcer)
	Agents get evidence
	Agents assess evidence
	Agents assess evidence announcer credibility
	Agents decide to:
	Revise prior beliefs based on evidence
	Ignore evidence and keep beliefs the same
	Seek advice (if available, create links)
	Revise assessments based on advice
	Revise prior beliefs or ignore advice and keep beliefs the same
	Tick (discrete time step, ends sequence of events)
*To go continuous (repeat sequence of events with initial conditions based on outcome of previous tick)*	Disconnect links
	Replace previous prior beliefs with revised beliefs
	Reset visibility (based on new prior beliefs)
	Reset credibility (based on new visibility)
	Announce evidence etc.

As mentioned previously, the single instance of advice giving/receiving does not necessarily indicate the presence of an opinion leader in a given context. The NOL-ABM is designed to view agent behaviors over time in order to examine the effect of changing individual beliefs on the need for and availability of opinion leaders. When the model is set to ‘continuous’ mode, the sequence of behaviors is repeated; however, the initial conditions are determined by the results of the previous run. Because of this, it is possible, for example, that based on the evidence, all of the nurses changed their beliefs such that they were no longer uncertain about new evidence and therefore would not need to ask for advice. Likewise, the advice givers may change their beliefs such that they themselves are no longer willing to act or give advice on the evidence. In this way, the ABM can be used to simulate a ‘time series,’ illustrating issues with dependence (prior beliefs) and the effect of the group characteristics on individual behavior.

### Model verification

ABM verification, the process by which agent-based models are shown to correspond to the underlying conceptual model is fundamental to the development of a rigorous model that can be validated and used to gain new insights about complex phenomena [21]. While verification is important for all models, guidelines for verifying ABM continue to evolve, and processes common in other forms of computational modeling are often used [8,19,21]. According to Rand and Rust [13], documentation, programmatic testing, and analysis of test cases are essential for ABM verification. Documentation of the preliminary NOL model, including the development of diagrams for use in programming, is detailed in Anderson and Whall [13]. Programmatic testing and exploratory analysis of simulated test case data are explained next.

### Iterative programmatic testing

The goal of programmatic verification is to reduce coding errors using various procedures for monitoring and ‘debugging’ the computer code [8,19,21]. Verification of the NOL-ABM occurred simultaneously with the iterative program development. For example, following the addition of each procedure, comparing computer-generated computations to hand-checked calculations resulted in coding adjustments. The identification of problems by continuously monitoring parameters reported on the model interface for irregularities (i.e. negative numbers or numbers outside the expected range) is another useful verification procedure. As the model development progressed, exporting simulation data into spreadsheets for analysis provided information that aided increasingly granular verification at the agent level. An example of ‘debugging’ occurred with the discovery that the procedure for ‘updating beliefs’ by replacing the ‘prior belief’ with the ‘new revised belief’ resulted in many nurses with new ‘prior beliefs’ with a score of zero. Tracking the code execution revealed that simply changing the procedure, so that only nurses who actually revised their beliefs replaced their ‘prior belief’ for the next ‘tick’, or sequence solved the problem.

### Exploratory analysis of simulated data

Once basic structural programming of the NOL model into the NOL-ABM was complete, the performance of systematic model exploration procedures was used as the next step toward verification of the NOL model. In order to verify that the NOL-ABM was capable of reproducing the conditions that affect the development of NOL according to the preliminary model, two types of simulation experiments were designed and executed for analysis. The first simulation procedure was ‘parameter sweeping’; it provides data about individual/agent variables under a variety of conditions. The second simulation focused on individual agent attributes over time in order to test the model’s representation of emerging opinion leaders.

### Parameter sweeping

Parameter sweeping is the process of systematically adjusting model input variables, such as the prior beliefs and the motives of the agents, in order to explore simulation outputs (e.g. the number of agents seeking or giving advice) using multiple combinations of possible conditions [8,19]. In order to explore potential differences, the design of the parameter sweep included simulation input values for the NOL-ABM variables (the number of staff nurses, number of educators, prior beliefs, motives, earned authority, evidence, and credibility threshold) purposely selected to enable comparisons among units with substantial differences (e.g. 50 nurse units compared with 200 nurse units). The simulation output variables were selected to verify that the NOL-ABM was capable of reasonably reproducing the proposed relationships. For example, by varying the prior beliefs and motives of the agents, the number of visible agents would be expected to differ since according to the preliminary NOL model, strong prior beliefs and pragmatic motives lead agents to act on their beliefs and become visible. Specified parameter selection for each of the above variables resulted in 288 possible combinations. Table 3 (left column) shows the details of the prescribed input parameter values and (right column) output variables included in the parameter sweep. For each of the 288 combinations, sequential model execution occurred 50 times (e.g. 14,400 model executions total). The selection of 50 iterations was based on balancing the need for replication with the practicalities of computer power. The resulting data were saved to a spreadsheet for analysis.

**Table 3:** **Parameters and data collection for simulation procedures**

**Parameter settings for input variables**	**Results reporting of output variables**
*Parameter sweep*	*Count agents:*
Number of nurses [50, 100, 200]^a^	Not visible
Number of educators [3, 7]	Visible
Mean prior belief [35, 65]	Available to give advice
Mean earned authority [35, 65]	Need advice
Mean motives [35, 65]	Gave advice
Mean evidence [40, 70]	Sought advice
Credibility threshold for giving advice [60, 70, 80]	Revised evidence assessment
Number of iterations [50]	Revised credibility assessment
	Revised beliefs
*Individual agent time series [initialization settings]*	*Variables at each time step*
Number of staff nurses [100]^b^	Prior belief (revised from previous step)
Number of educators [5]^b^	Evidence (newly introduced each step)
Mean prior belief [50]	Credibility (changes based on new beliefs)
Mean earned authority [50]	Assessed evidence
Mean motives [50]	Assessed credibility
Mean evidence [60]	Available to give advice
Credibility threshold for giving advice [65]^b^	Need advice
Number of iterations [20]	Gave advice
	Sought advice
	Revised beliefs based evidence
	Revised beliefs based on advice

^a^Numbers indicate the values of the variables used in the simulation procedures.

^b^Variables held static for each of 20 model executions.

### Individual agent time series

The attributes of individual agents are the focus of the second simulation procedure used to verify the NOL model. The simulation experiment was devised to examine the agents over time in order to explore the effect of changing individual beliefs, based on the introduction of new evidence, on the emergence of opinion leaders (consistently available to give advice + sought out for advice) on a unit. In this simulation, the number of staff nurses, number of educators, and credibility threshold were static for each of 20 model executions (Table 3; bottom half). Upon initialization, each individual agent was assigned a unique value for prior belief, earned authority, and motives—all attributes of individual agents (values 1–100 around a preset mean and standard deviation). Although each individual agents’ earned authority and motives remained constant over the time series, their beliefs were (potentially) revised, based on the evidence and any advice they received at each time step. According to the preliminary model, changing beliefs may affect an agent’s degree of uncertainty and need for advice as well as the availability of other agents to be available for advice giving (the opinion leaders). The parameter values for the initialization settings were selected to represent an ‘average’ unit based on the range of possible values. The output variables were measures of agent evidence and credibility assessments and their behaviors related to advice. The simulated data were collected and exported to a spreadsheet for statistical analysis and visualization. The list of model input and output variables for this individual agent time series are shown in the lower half of Table 3.

## Results

### Results of the parameter sweep

Following the model execution, the raw data were analyzed descriptively in order to obtain, for each of the 288 possible combinations of variables, the minimum, maximum, and mean for each individual agent variable included in the 50 model runs. The aggregated descriptive results for the *N* = 288 possible simulation scenarios are shown in Table 4. The following results are based on the data set created from the means of each variable.

**Table 4:** **Descriptive statistical results of the parameter sweep**

**Individual agent variables**	**Minimum**	**Maximum**	**Mean**	**Standard deviation**
Not visible	9	203	74.0	51.14
Visible	0	191	48.6	43.57
Available	0	88	7.8	13.20
Number who give advice	0	77	7.0	11.83
Number who need advice	1	107	23.5	23.03
Number who seek advice if available	0	91	13.5	17.45
Credibility	38	63	50.4	7.47
Prior belief	34	72	53.3	12.16
Number with new beliefs	11	176	70.1	43.54
Number with revised evidence assessment	0	79	6.0	12.98
Number with revised credibility assessment	0	56	9.1	11.68
Number with revised beliefs	9	58	30.5	11.26

According to the preliminary NOL model, individuals must be visible in order to be available as a resource for others who are seeking another’s opinion about new evidence. Individuals become visible when they act on the strength of their beliefs. In addition, a person’s motives influence visibility by changing the threshold for actions; those with pragmatic motives are more likely to act at a lower threshold of belief [13]. In order to verify that the NOL-ABM was performing consistent with posited relationships in the preliminary NOL model [10], we first tested the effect of prior beliefs and motives on the dependent variable of visibility. Regression results shown in Table 5, (row A) confirm that the NOL-ABM performs as planned; that is as prior beliefs and motives predict visibility. The results also confirm that pragmatic motives (e.g. value <50) and higher prior beliefs have a positive association, whereas epistemic motives (≥50) and low prior beliefs are inversely associated with agent visibility.

**Table 5:** **Regression results of the parameter sweep**

**Independent variable**	**B**	**SE**	**β**	**Significance**	**df**	**F**	**Significance**	**R square**	**Adjusted R square**	**SE**
*A. An agent’s motives and prior beliefs predict his visibility*										
Prior beliefs	1.410	.149	.486	<.001	2	47.999	<.001	.252	.247	37.812
Motives	−.362	.149	−.125	.016						
*B. An agent’s visibility and authority predict his credibility*										
Earned authority	.474	.008	.953	<.001	2	1623.452	<.001	.919	.919	2.129
Visibility	−.014	.003	−.081	<.001						
*C. An agent’s prior beliefs and the evidence predict his new beliefs*										
Prior beliefs	.192	.046	.207	<.001	2	98.901	<.001	.410	.406	8.683
New evidence	.404	.038	.528	<.001						
*D. The number of visible agents and the credibility threshold of the unit predict the number of agents available to give advice*										
Visibility	.142	.015	.469	<.001	2	70.898	<.001	.332	.328	10.801
Credibility threshold	−.519	.078	−.322	<.001						
*E. The agents’ prior beliefs, the probability of the new evidence and the evidence announcer credibility predict the number of agents who will need advice*										
Prior beliefs	.178	.094	.094	.060	3	67.291	<.001	.415	.409	17.701
New evidence	−.078	.078	−.050	.316						
Announcer credibility	−1.944	.140	−.634	<.001						
*F. Agents who revise their assessment of the evidence and the credibility of the evidence announcer based on advice revise their beliefs*										
Number of agents with revised evidence assessment	−.080	.193	−.024	.678	2	36.188	<.001	.203	.197	39.016
Number of agents with revised assessment of announcer credibility	1.711	.215	.459	<.001						
*G. When agents who need advice receive it, the number of people with revised beliefs is predicted*										
Give advice	1.059	.206	.288	<.001	2	22.001	<.001	.134	.128	40.664
Need advice	.532	.106	.282	<.001						

The preliminary NOL model posits that the development of opinion leaders depends on the availability of individuals able to perform the role (visibility and credibility) as well as other individuals who are uncertain and need advice (Table 5, rows B and C) [13]. According to the NOL-ABM, agent visibility and the credibility threshold on a unit predict availability, as expected (Table 5, row D). The idea that units that have a higher credibility threshold also have fewer agents available to act as potential opinion leaders is illustrated by the inverse relationship among these two variables (Table 5, row D). It is proposed that agent uncertainty is based on a threshold of evidence and credibility assessments performed by the individual agent (Table 5, rows E and F). When agents are able to get the advice they need, they are able to revise their beliefs accordingly (Table 5- Row G). Again, results of regression analysis of the model output confirm that the NOL-ABM is performing as intended and supports these relationships in the NOL model [10].

In all of the 288 combinations of variables specified for the parameter sweep, the results show that at least a few of the agents needed advice (e.g. another opinion) to make a decision about the evidence due to uncertainty. In 73 of the different parameter combinations, there were no opinion givers available. Logistic regression was used to analyze the characteristics that would affect the likelihood of available opinion leaders. The results show several independent variables that predict availability. These include the prior beliefs of the agents (*p* < .0005), earned authority (*p* < .0005), required credibility (*p* < .0005), number of nurses (*p* = .001), and the evidence (*p* = .028). Differences in the motives (*p* = .304) and the number of educators (*p* = .377) were not significant.

### Results of the individual agent time series

The analysis of the simulated data output from the time series NOL-ABM experiment was primarily descriptive since the aim was to verify consistency with the preliminary model. Frequency tables, manually checking calculations, and graphic visualization were used in this phase of analysis for model verification.

**Figure 3:**
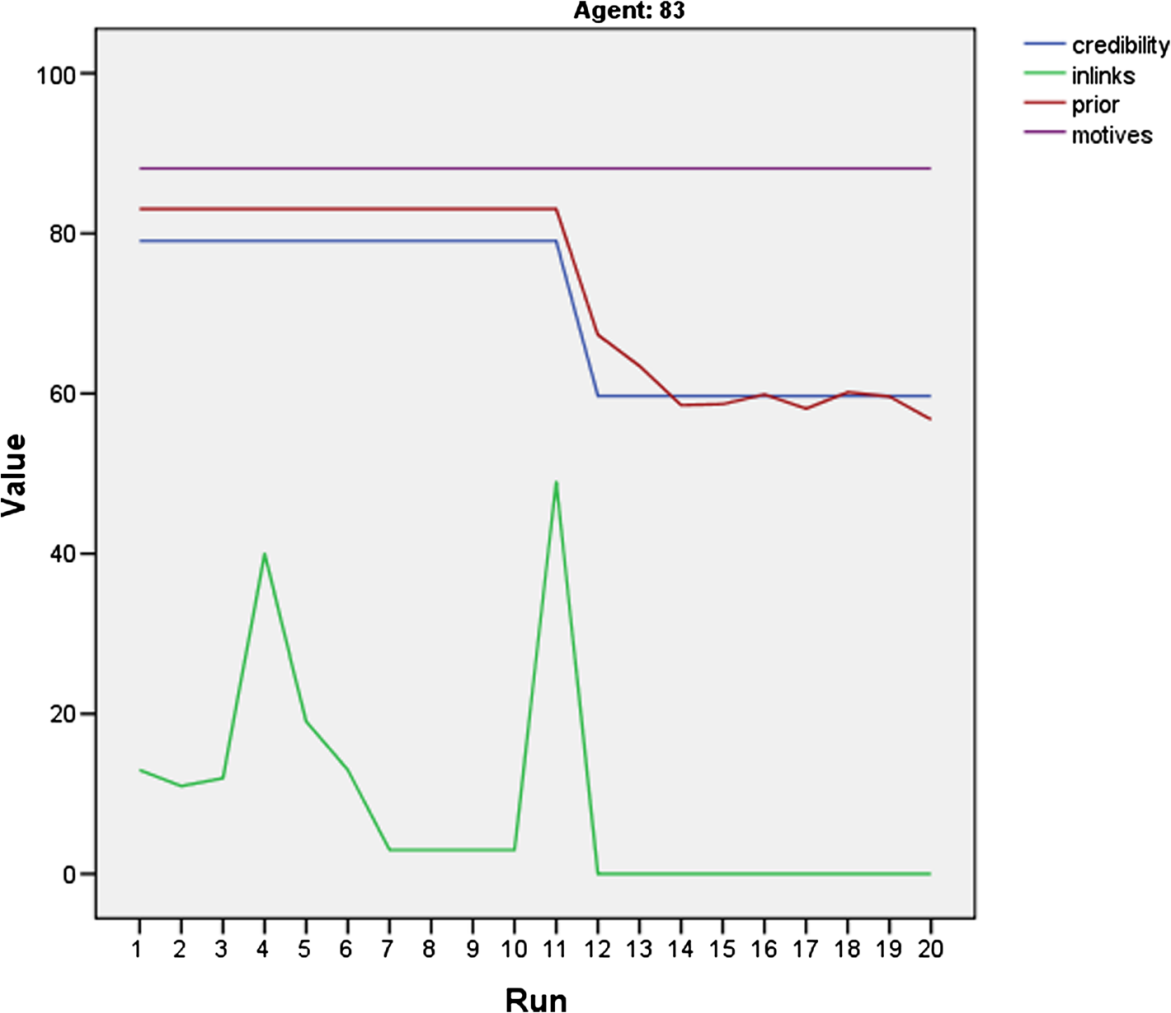
**Availability of an agent to give advice over time.** This figure illustrates the results of the time series data for one specific agent. When agent 83’s prior beliefs change, based on new evidence, she is no longer confident enough to act on her beliefs. This change in visibility reduces the agent’s credibility and thus results in a lack of contact by advice seekers, shown by the lack of in-links following this change.

First, individual agents who gave advice (e.g. had at least one in-link from other agents who were uncertain) were tracked over time to evaluate whether the same individuals had in-links at each time point. Four nurses (of the 106 total ‘subjects’) had in-links over the course of the 20 time points. None of these nurses was available to opinion seekers at every time point and one of them was available for giving advice only once. The variation in availability is the result of changes in the individual agents’ beliefs or credibility. This is explained by the preliminary NOL model, which posits that an individual’s change in beliefs is the result of revision based on new evidence and can affect visibility, depending on the motives of the individual. Changes in credibility may be affected by visibility. This is because the credibility assessment calculation of nurses with visible beliefs weights more heavily toward the earned authority than the unearned authority (which is assigned based on the job title) (Table 1). Figure 3 shows an example of the relationships between motives, prior beliefs, credibility, and in-links, over time, for one nurse agent. Since nurse agent no. 83 has a ‘motives’ score of 88, indicating epistemic motives, the nurse will act on prior beliefs greater than 70, a relatively high threshold for action. Since the credibility threshold for the other nurses seeking an opinion is set at 65 for this simulation, nurse agent no. 83 is both credible and visible, and therefore receives in-links from other nurses who need advice.

After a belief revision during time-period 11, nurse agent no. 83’s prior belief drops below the visibility threshold. This change in visibility affects the credibility since when the nurse agents on the unit are unaware of nurse agent no. 83’s beliefs (because she is no longer acting on them); they give more weight to the unearned authority, an agent-set variable that equals 50 because agent no. 83 is a staff nurse and not an educator or manager. This is in contrast to the previous time periods that in which credibility was based more on the earned authority, which is grounded on individual performance. This example provides evidence that the NOL-ABM is performing coded procedures appropriately.

Opinion seekers may revise their beliefs based on the information obtained from the advice giver. In this 20 run time series, all of the opinion seekers (47 agents with out-links) were uncertain about the credibility of the evidence announcer based on their individual results of the coded assessment procedure. In addition, 11 of these nurse agents were also uncertain about the strength of the evidence. Like the nurse agents with in-links, those agents who sought advice tended to display this characteristic over time; however, 20 of the 47 sought advice only twice. In several instances, the revised assessment of the evidence based on the second opinion resulted in a decision to ignore the evidence.

### Overall summary of results

The construction of the NOL-ABM presented here represents the basic structure of a model of opinion leadership among nurses and in the context of nursing practice. Iterative development and verification testing resulted in a dynamic agent-based model capable of producing simulation results consistent with the preliminary NOL model developed in phase 1 of this research [13]. Parameter sweep and individual results indicate that nurses revise their beliefs based on their previous opinions and their assessment of new evidence. Sometimes, the new evidence is of questionable credibility and is either ignored or further explored by seeking the opinions of credible colleagues.

The simulation results also show that nurse agents act on their strength of beliefs and, as a result, become an opinion resource for their uncertain colleagues, depending on their perceived credibility. Over time, a few individual agents consistently act as this type of resource and have the potential to become opinion leaders—those individuals who frequently influence the opinions of others when they are sought out for advice. Analysis indicates opinion leaders are more likely to emerge on units in which there are credible nurses with strong beliefs, available to act as resources for other nurse agents who are uncertain about new evidence. The degree to which the nursing unit consists of both uncertain staff members and others willing and able to share their opinions has implications for predicting the usefulness of an opinion leader strategy for improving the adoption of evidence-based nursing practice. For example, on a unit composed primarily of novice nurses, there may be a lack of credible resource persons. On a unit of mostly expert nurses, with strong beliefs, the credibility threshold may be so high that it prevents any reliance on the opinion of colleagues when making decisions about revising beliefs based on new evidence.

## Discussion

Our results show that the NOL-ABM is capable of representing the effect of an opinion leader on another person’s decision-making when it comes to adopting new evidence. As a representation of the preliminary NOL model [13], the ABM is a partial model that does not include some of the more detailed aspects of variables in the original model, for example, more specific attributes about the new evidence. According to Miller and Page [22], the process of developing and interacting with a computational model often leads the theorist to discover new insights and avenues for further development of the model. Several priorities for model extension and increased specificity were identified, based on the development and verification results described here.

First, increasing the dimensionality by adding more details to the nurse agents and the characteristics of the evidence are priorities for enhancing the correspondence of the model with real world situations and increasing its usefulness. Increased specificity of the agents (for example, using real-world data to set parameters) and the evidence will contribute to further process refinements related to credibility and belief revision. In terms of increasing agent heterogeneity, characteristics such as level of education, age, years of experience, and shifts worked are examples of factors that could provide diversity. Adding other types of agents, such as physicians or hospital administrators, would add another level of complexity that has potential for differentiating how opinion leadership differs among professional groups. Expanding the social networks of the agents is also a priority. In this iteration of the NOL-ABM, it is assumed that all of the agents know each other equally well. Variations based on length of acquaintance, professional group affiliation, job category and the addition of one, or more, different nursing units may improve the explanatory power of the model. In addition, these factors may affect credibility and access to evidence and could therefore contribute to strengthening the representation of these processes.

The development of the preliminary NOL model involved a process of theory derivation using rational belief revision as a basis for explaining individual and group opinion formation and the emergence of opinion leaders over time. Epstein’s [11] pioneering work developing ‘Agent_Zero,’ based on neuro-cognitive foundations and theories of associative learning, suggests avenues for future development through the use of coded ‘modules.’ For example, the creation, by Epstein, of agents that include an affective component, in addition to the cognitive and social component resulted in agents that may be more realistic than rational thinkers lacking in emotion. The NOL-ABM could potentially be extended to include all or parts of Epstein’s affective module. [11] Likewise, computer-coded modules (empirically or theoretically based) designed to represent other cognitive mechanisms could be substituted for ‘rational belief’ (e.g. bounded rationality). Combining model features or comparison of outcomes generated by multiple models in validation studies advance the development of usable models [11].

Because of the key role of subjective credibility assessment in the evaluation of evidence and the emergence of opinion leaders, refining the process of credibility assessment is also a priority for model improvement and extension. For example, personal authority, based on characteristics such as friendship ties, or prestige is another potential component of credibility assessment [17]. Other network effects, such as the strength of relationships, formation of cliques, and connections, or lack of connections, between groups may contribute to the ability of agents to assess each other’s credibility or affect the accuracy of the assessment. By adding some complexity to the social environment, using empirical data, an enhanced understanding of the conditions conducive to opinion leader emergence and stability over time may be possible.

The process of credibility assessment is important not only for the identification of potential opinion leaders, but also for determining an agent’s perceived need for another opinion which becomes apparent when the agent assesses the new evidence. Areas for improved NOL-ABM development in this area include incorporating agent preferences for which particular agent’s opinion they may seek and to what degree the new opinion influences the agent’s assessment. Additionally, potential improvements to aspects of the new evidence and the evidence announcement procedure were identified. These include allowing individual agents to act as intermediaries for bringing new evidence to other groups or having agents consider the weight of the evidence when revising their beliefs. The agent behaviors related to uncertainty and credibility assessment suggest that thresholds are key mechanisms that guide actions.

Although normative models such as the NOL-ABM can provide useful insight for understanding phenomenon such as NOL, an important advantage of using ABM as a theory development tool is that it is possible for the model developer to include actual empirical data when programming variables. For example, results from Li et al.’s study about reducing HIV stigma showed that opinion leaders had higher scores on a scale of message diffusion among their peers and co-workers [23,24]. Instead of assigning random values to the model variables, the use of the empirical mean and standard deviation obtained from the study measures related to message diffusion could be used to set new ABM parameters. Incorporating new concepts into the model as an extension is one way to develop understanding about the process of message delivery. In addition to using ‘hybrid’ models, containing both real and simulated values, for exploration and scenario predictions, the comparison of simulated and empirical data can lead to improved theoretical explanation and knowledge discovery and contributes to model validation. ABM and simulation can potentially highlight the need for empirical data collection that would not be evident without the use of a theoretical model [9].

## Conclusions

ABM is a theory development tool that shows promise for use in nursing and health care, particularly for representing multi-level, context sensitive, and dynamic phenomena such as opinion leadership. The NOL-ABM development and verification processes described here are a first step. We have shown that the ABM corresponds to the underlying conceptual model. Beginning ABM development with a clear understanding of the essential conceptual attributes and processes provides a framework and basic structure for model development. The process of continued, iterative model extensions, such as those described above, enables the development of a model that provides just enough detail for theoretical explanation without introducing extraneous variables that add unnecessary complexity [22]. The next phase of model development includes combining extensions or revisions with ongoing verification and establishing a process for model validation procedures, including use of empirical data in order to test the ABMs usefulness for explaining real world phenomena. Examples of validation methods include simulation or empirical research studies designed to answer research questions about opinion leadership. For instance, do individuals assign greater weight to the strength of the evidence or the credibility of the messenger? In multidisciplinary settings, are there differences in how individuals attribute credibility and what are the factors involved? These and other questions for future research will contribute to the overall aim of developing of a workable, dynamic representation of opinion leadership in complex healthcare environments.
